# Are we running out of fish? Fish, health and sustainability

**DOI:** 10.1017/S1368980023001544

**Published:** 2023-10

**Authors:** Aditya Khetan, Vittal Hejjaji, Richard Josephson

**Affiliations:** 1 Division of Cardiology, Department of Medicine, McMaster University, Hamilton, ON, Canada; 2 Saint Luke’s Mid America Heart Institute/University of Missouri, Kansas City, Missouri, USA; 3 Harrington Heart and Vascular Institute, Case Western Reserve University, Cleveland, OH, USA

**Keywords:** Seafood, Sustainability, Aquaculture, Fish stocks, Health

‘I believe that the cod fishery, the herring fishery, the pilchard fishery, the mackerel fishery, and probably all the great sea fisheries, are inexhaustible; that is to say, that nothing we do seriously affects the number of the fish’.

T.H. Huxley, Biologist, 1883

The summer of 1992 started like just another summer in the Canadian province of Newfoundland. For the last 500 years, fishing had shaped the region’s life and culture, like it did for much of Atlantic Canada. In Newfoundland, an estimated 30 000 people were getting ready for another summer of work in the fishing industry, when their lives changed permanently. The Atlantic Cod mass had collapsed to 7 % of earlier levels, leading to the Federal government announcing a moratorium on the Northern Cod fishery in July 1992. 500 years of history came to a standstill, sparking widespread unemployment, emigration and socio-economic changes in Newfoundland.

There are multiple reasons for the collapse of the Atlantic Cod fishery, including technology (that increased the volume of fish caught), mismanagement and poor understanding of the area’s ecosystem. However, what happened with the Atlantic Cod fishery is not an exception. In fact, it may be a sign of the coming times. Between 1990 and 2017, as total fish consumption globally rose by 122 %, the percentage of fish stocks within biologically sustainable levels dropped from 90 % to 65·8 %^(1)^. Furthermore, we are running out of underfished stocks, which have decreased from just over 25 % in 1990 to 6·2 % in 2017^(1)^. In 2005, an estimate found that if current rates of fishing continue, global fishing would completely collapse by 2048^(2)^. An updated analysis in 2016 showed little progress, confirming that the average state of global fish stocks is not only poor but also continues to decline^(3)^.

Of the 158 million tonnes of seafood produced in 2019, 72 % was consumed in Asia, while its population represented 60 % of the world population. China, by far the largest consumer, alone accounted for 36 % of global consumption. Other large consumers include Indonesia, India, USA and Japan, which together consumed 23 % of global seafood^(4)^. On a per capita basis, outside Asia, countries with the largest seafood footprint include Iceland, Portugal, Norway and Spain^(5)^.

Despite the increase in global fish consumption and decline in global fish stocks, guidelines suggest individuals are not consuming enough fish for their optimal health. For instance, in the USA (the largest consumer of fish outside Asia by tonnage), the average seafood consumption is estimated at 100 g (3·5 ounces) per week, as opposed to the recommended 225 g (8 ounces) per week^(6)^. For a healthy Mediterranean style eating pattern, the recommendation is to consume up to 425 g (15 ounces) per week^(7)^. It is worth noting that in 2017, at a global level, the Mediterranean region had the highest percentage (62·5 %) of stocks fished at unsustainable levels^(1)^. The scientific report of the 2015 dietary guidelines advisory committee in the USA acknowledges issues with sustainability of fish stocks but suggests that ‘expanding farm-raised seafood (aquaculture) has the potential to ensure sufficient amounts of seafood to allow the U.S. population to consume levels recommended by dietary guidelines’^(8)^. However, this optimistic outlook is a conjecture and does not acknowledge the limitations of aquaculture. Aquaculture is subject to resource limitations, causes pollution (similar to industrial animal food production), deforestation, loss of wetlands and has considerable social and ecological impacts, leading to limited expansion of aquaculture in wealthy democratic countries^(9)^. Top global importers of seafood are therefore largely wealthy countries, led by the USA, followed by China, Japan, France and Germany^(10)^. Given that countries such as the USA import around 65–70 % of its seafood, it is possible that a continued focus on aquaculture to meet seafood demand, in the face of dwindling global wild fish stocks, will exacerbate global food inequities and ‘outsource’ aquaculture pollution, other associated costs and negative externalities to nutritionally vulnerable nations^(11,12)^. Many nutritionally or environmentally vulnerable nations are already among the top exporters of seafood (India, Thailand, Ecuador and Indonesia)^(10)^. While there is room for improvement in aquaculture governance, which can lead to improved supplies in the short term, ecological limits will be met eventually if demand for seafood continues to rise at current levels. Given the intrinsic feed conversion inefficiencies in animal-based protein, the idea of ecological limits to seafood production is consistent with the more widely known ecological limits of meat production.

**Fig. 1:**
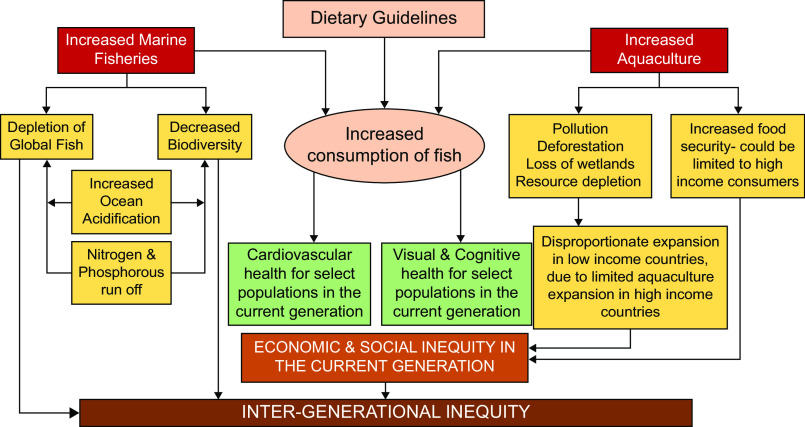
Conceptual model of potential effects of current dietary guidelines, along with current practices of fish production and consumption

So how do we make dietary recommendations that incorporate issues of sustainability in the marine food system? A good first step would be to acknowledge the issue and avoid overly optimistic solutions that fail to incorporate the ‘wicked’ nature of the problem^(13)^. Currently, only four out of eighty-three countries (Sweden, Germany, Brazil and Qatar) that have dietary guidelines mention sustainability^(14)^. Guidelines that incorporate sustainability mention the critical state of wild fisheries and the negative impacts of aquaculture. Nevertheless, they continue to recommend consuming fish in quantities consistent with health recommendations. This is a trade-off that will not go away and needs addressing, necessitating a second step. Taking a systems approach to fish that simultaneously considers environmental, social and economic outcomes, in addition to health outcomes, will allow a conceptual model that enhances our understanding of the trade-offs involved (Figure 1). A system-based approach will allow the development of recommendations that balance competing priorities and acknowledge the interdependence of health outcomes with economic, social and environmental outcomes. A third step is clearly acknowledging that a healthy eating pattern does not necessarily have to incorporate seafood. Highlighting a vegetarian eating pattern as an example of a healthy eating pattern, as in the 2020 dietary guidelines for Americans, is a good step in this direction. Going further, creating recommendations that guide people on pursuing healthy eating patterns without seafood can provide a clear roadmap for people who want to reduce their seafood intake to enhance the sustainability of their diet. In addition, guidelines should highlight seafood that have high nutritional value at low emissions, such as wild-caught small pelagic and salmonid species, and farmed bivalves like mussels and oysters^(15)^. With increasing awareness of the dependence of human health on planetary health, sustainability is a growing factor in the dietary choices of individuals, particularly younger individuals who stand to lose the most from our current unsustainable practices. However, this progress has happened in spite of our dietary recommendations, not because of it. It is time that dietary guidelines become a driver of sustainable changes that can improve the welfare of not just our generation but of many generations to come. The next version of country-level dietary guidelines, around the world, presents an opportunity for health professionals and nutritionists to lead the change.
